# Mid-term outcome (11–90 months) of the extensor retinaculum flap procedure for extensor carpi ulnaris tendon instability

**DOI:** 10.1007/s00402-019-03227-2

**Published:** 2019-06-20

**Authors:** Kaiser Peter, Haug Luzian, Gabl Markus, Rudisch Ansgar, Klauser Andrea, Rohit Arora

**Affiliations:** 1 Department of Trauma Surgery, Medical University of Innsbruck, Anichstr. 35, 6020 Innsbruck, Austria; 2 Department for Plastic, Reconstructive and Hand Surgery, Inselspital, Bern University Hospital, Bern, Switzerland; 3 Department of Radiology, Medical University of Innsbruck, Innsbruck, Austria

**Keywords:** ECU, Tendon, Instability, Retinaculum, Sub-sheath, Treatment, Wrist pain

## Abstract

**Introduction:**

The aim of our study was the assessment of the mid-term outcome of patients treated with a pediculated extensor retinaculum flap for extensor carpi ulnaris (ECU) tendon subluxation including postoperative tendon stability control.

**Materials and methods:**

Twelve patients treated with an extensor retinaculum flap for symptomatic ECU tendon instability were retrospectively evaluated. Follow-up examinations included functional and radiologic assessment. The range of motion, grip strength, DASH score, PRWE score, Krimmer score and subjective satisfaction were recorded. A rotation-movie MRI was conducted before and after surgery to visualize tendon displacement.

**Results:**

Wrist extension was 65.8° (SD 10.0°), flexion 64.2° (SD 12.2°), radial deviation 15.8° (SD 6.0°), ulnar deviation 32.1° (SD 7.2°), pronation 82.5° (SD 9.4°) and supination 85.0° (SD 9.0°). Mean grip strength was 30.5 kg (SD 8.9 kg). Six patients presented an excellent, four a good, one a fair and one a poor result on the Krimmer score. The DASH and PRWE scores showed a mean of 24.2 (SD 25.1) and 32.2 (SD 29.4) points.

MRI showed a dislocation (*n* = 7) or subluxation (*n* = 5) of the ECU tendon preoperatively. Five patients showed an unchanged displacement pattern postoperatively.

**Conclusion:**

The pediculated extensor retinaculum flap as a treatment for a symptomatic ECU instability shows good to excellent results and a high subjective satisfaction independent of postoperative ECU tendon displacement

## Introduction

Extensor carpi ulnaris (ECU) tendon dislocation or subluxation can be one cause of ulnar-sided wrist pain. The reason for displacement is either an injury to the tendon sub-sheath caused by trauma or rheumatic genesis [1, 2].

The extensor carpi ulnaris tendon is enclosed in an independent osteofibrous tunnel and stabilized by its sub-sheath. It prevents the tendon from subluxation out of the ulnar groove during a pronation–supination motion. Diagnosis of subluxation or dislocation is made clinically and confirmed with ultrasound or MRI [3–6].

Acute injuries can be treated conservatively with an upper-arm cast and the forearm mainly in pronation, wrist extension and radial abduction [7–10]. Surgical treatment is necessary for patients suffering from chronic ECU tendon dislocation or subluxation and persistent pain.

Several surgical procedures have been described. Some authors prefer a direct repair of the tendon sheath [8, 11, 12], others a reconstruction using a pediculated [11, 13] or a free [11, 14, 15] strip of the extensor retinaculum or the fascia lata [16] and others suggested tendon sheath reconstruction subperiosteally and groove deepening [3] or a complete release of the 6th compartment [17].

Reporting on the outcome of surgical treatment is scarce. The existing literature is limited to case report studies that describe surgical treatment in a limited numbers of patients without consistent outcome parameters and with a variable follow-up. Moreover, there are no investigations on the objective, postoperative position and displacement of the ECU tendon.

The aim of our study was, therefore, the assessment of the mid-term outcome of patients treated with a pediculated extensor retinaculum flap for ECU tendon subluxation.

## Materials and methods

A retrospective evaluation was conducted on 12 consecutive patients (10 female and 2 male; mean age 35.3 years (19–53 years); affected right side 8 times; affected dominant side 9 times) who were treated surgically with an extensor retinaculum flap described by Spinner and Kaplan [13] for ECU tendon dislocation between 2003 and 2010. Two patients were excluded because they were not available for a follow-up. The study was approved by the institutional review board including patient’s informed consent.

All patients suffered from ulnar-sided wrist pain with a clinical instability of the ECU tendon in maximal supination, flexion and ulnar deviation of the wrist. None of the patients responded to conservative treatment including pain medication, immobilization and physical therapy.

Six patients had a single ECU tendon instability while the other six patients had concomitant injuries including degeneration of the triangular fibrocartilage complex (TFCC) (3 cases), carpal instability (1 case), ulnocarpal impingement (1 case), and distal radius fracture (2 cases). The latter patients had additional treatment including arthroscopy, shaving of the TFCC (TFCC lesion), shrinking of the capsule (carpal instability), and shortening of the processus styloideus ulnae (ulnocarpal impingement).

ECU tendon instability and surgical indication was confirmed with a rotation-movie MRI of the injured wrist [6] and classified according to Inoue and Tamura [11]. Images were acquired with a 1.5 T MRI (Magentom, Avanto, Siemens, Germany) in real time (movements from maximum pronation to maximum supination) using an axial true FISP sequence (slice thickness: 6 mm, TR/TE: 4/1.55 ms, FA 40°, acquisition matrix: 208 × 133, voxel-size 0.74 × 0.74 × 6 mm^3^). The MRI images were evaluated by a radiologist, who is highly experienced in musculoskeletal imaging. A displacement of 100% of the ECU tendon’s width beyond the ulnar border of the ulnar groove represented an ECU tendon dislocation while a partial displacement of the tendon’s width represented a subluxation.

Follow-up examinations were conducted at an average of 44.9 months (11–90 months) after surgery and included a functional and radiologic assessment.

The active range of wrist extension, flexion, pronation and supination was assessed using a goniometer. Grip power was measured with a hand dynamometer (Jamar, FEI, Irvington, NY, USA). Results were compared to the opposite, non-injured side.

Functional subjective outcomes were measured using the disability of the arm, shoulder, and hand (DASH) questionnaire and the patient-rated wrist evaluation (PRWE) score. Wrist pain was evaluated using the visual analogue scale (0 = no pain; 10 = severe pain). Surgical results were scored using the Krimmer wrist score (80–100 points = excellent, 65–80 points = good, 50–65 points = fair, 0–50 points = poor). Patients scored their subjective satisfaction with the injured wrist after surgery on a scale of 1–10 (1 corresponded with the lowest satisfaction and 10 indicated full satisfaction).

**Fig. 1:**
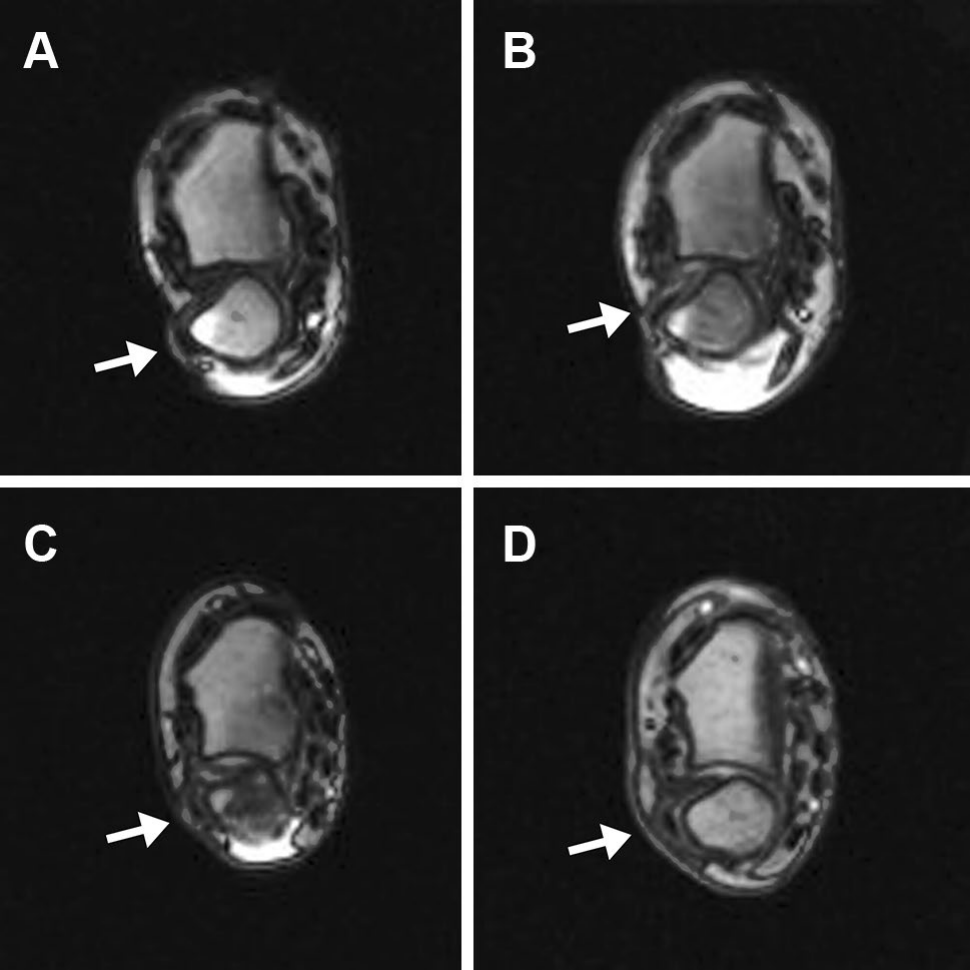
Rotation-movie MRI visualizing ECU tendon displacement (arrow) in full supination: ECU tendon dislocation before surgery (**a**) and corrected ECU position postoperatively without dislocation (**b**); ECU tendon dislocation before surgery (**c**) and persisting ECU tendon dislocation postoperatively (**d**)

Postoperative radiologic assessment consisted of a rotation-movie MRI for tendon displacement visualization (Fig. 1).

Results are presented using descriptive statistics.

### Surgical technique

**Fig. 2:**
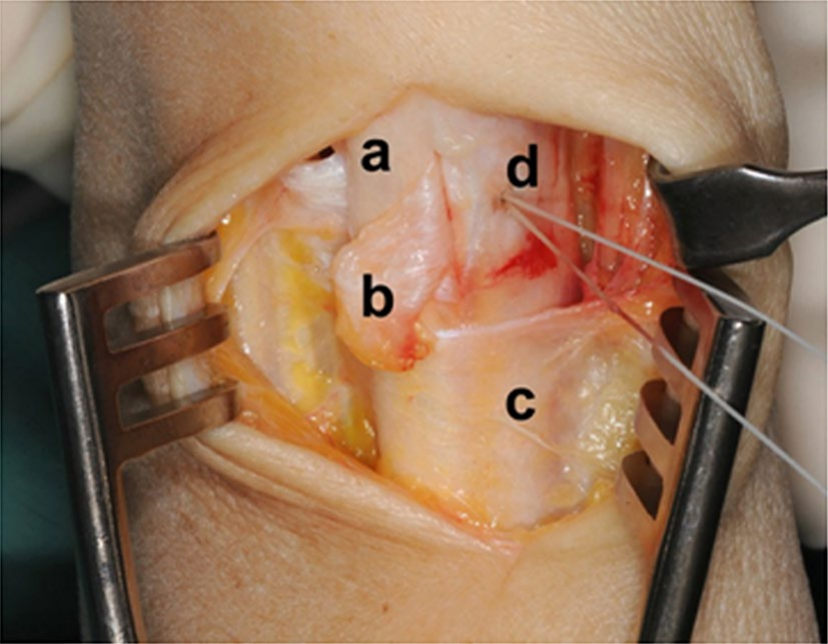
Intraoperative view: **a** ECU tendon, **b** retinaculum flap, **c** extensor retinaculum, **d** anchor fixation at the top of the ulnar border of groove

**Fig. 3:**
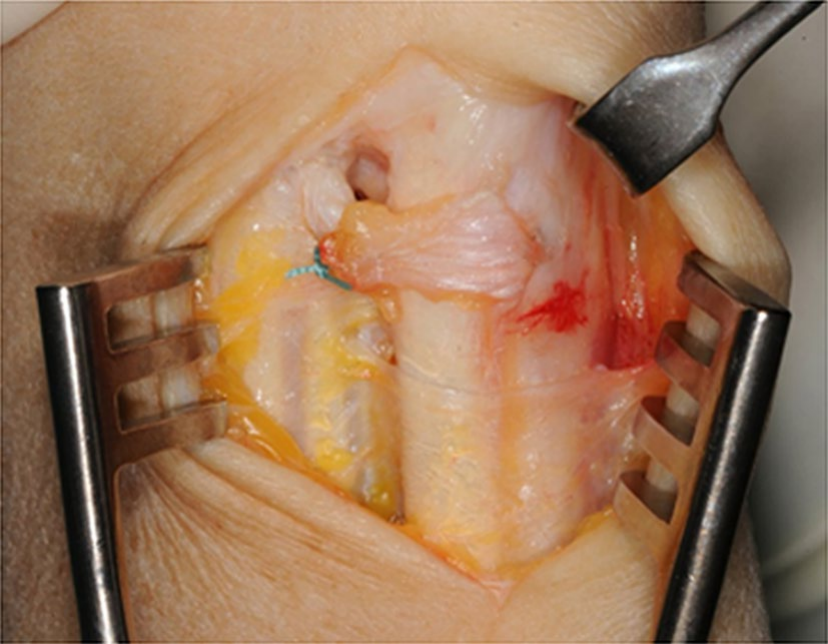
Intraoperative view: retinaculum flap looped around the ECU tendon

The sixth extensor compartment was accessed using a dorsoulnar approach. The sensory branches of the ulnar nerve were recognized and protected. The extensor retinaculum was opened over the sixth extensor compartment. A small radially based flap was isolated and passed underneath the ECU tendon. This retinaculum flap was then attached ulnarly using trans-osseous sutures at the top of the ulnar border of groove to prevent its movement (Fig. 2). Then the flap was looped around the ECU tendon and reattached at its basis to itself (Fig. 3). Immobilization in an upper-arm sugar tongue cast was conducted for 6 weeks with restriction for pro- and supination while elbow extension flexion was permitted. After brace removal, physical therapy was started.

## Results

Patient’s demographics and results are presented in Table 1.

**Table 1:** Summary of cases

*n*	Age (years)	Follow-up (months)	Affected dominant side	Inoue and Tamara classification [11]	Injury occurrence	Additional injury	DASH/PRWE score	Subjective satisfaction	MRI preop^a^	MRI postop^a^	Krimmer scoring scale
1	27	13	Yes	A	Tennis	–	12/9	9	+	–	Good
2	27	90	No	A	n/a	–	0/0	10	+ +	+ +	Excellent
3	44	24	Yes	C	Distorsion	–	9/12	9	+	–	Excellent
4	50	94	Yes	A	n/a	TFCC	18/21	8	+ +	+ +	Excellent
5	32	15	Yes	B	n/a	CI	65/66	8	+ +	–	Good
6	53	58	Yes	C	Fall	TFCC	77/92	1	+	–	Poor
7	31	60	No	C	Fall	UI	2/5	9	+	n/a	Excellent
8	41	34	Yes	C	Fall	DRF	15/55	9	+	+	Excellent
9	34	24	No	A	Fall	TFCC, DRF	34/48	8	+ +	+ +	Good
10	24	40	Yes	A	n/a	–	3/2	10	+ +	+ +	Excellent
11	41	11	Yes	C	n/a	–	42/48	6	+ +	–	Fair
12	19	76	Yes	n/a	n/a	–	14/30	6	+ +	–	Good

*TFCC* lesion of the triangular fibrocartilage complex, *CI* carpal instability, *UI* ulnocarpal impingement, *DRF* distal radius fracture, *n/a* not assessable

^a^( +  + ) equals ECU tendon dislocation over the ulnar margin of the groove ( + ) equals ECU tendon subluxation (–) equals no dislocation or subluxation of the ECU tendon

Wrist extension was 65.8° (SD 10.0°), flexion 64.2° (SD 12.2°), radial deviation 15.8° (SD 6.0°), ulnar deviation 32.1° (SD 7.2°), pronation 82.5° (SD 9.4°) and supination 85.0° (SD 9.0°), respectively, extension was 95.% (SD 12%), flexion 89.7% (SD 14.4%), radial deviation 72.8% (SD 28.7%), ulnar deviation 87.4% (SD 29.1%), pronation 94.0% (SD 11.0%) and supination 96.9% (SD 6.4%) compared to the uninjured side. The mean grip strength was 30.5 kg (SD 8.9 kg) which resembles 92.1% (SD 22.7%) compared to the uninjured side. Six patients presented an excellent, four a good, one a fair and one a poor result according to the Krimmer scoring scale (Table 2).

**Table 2:** Range of motion and grip strength of all cases for the postoperative injured side

*n*	Extension	Flexion	Radial deviation	Ulnar deviation	Pronation	Supination	Grip strength^a^
1	60°	75°	10°	30°	80°	90°	18 kg/56%
2	80°	65°	20°	35°	85°	90°	34 kg/92%
3	65°	65°	20°	40°	80°	80°	28 kg/108%
4	70°	70°	20°	40°	90°	80°	39 kg/134%
5	55°	60°	15°	20°	85°	90°	26 kg/84%
6	45°	40°	0°	30°	60°	90°	16 kg/67%
7	60°	70°	15°	40°	80°	80°	n/a
8	75°	55°	20°	30°	70°	90°	33 kg/100%
9	65°	60°	15°	30°	90°	90°	28 kg/72%
10	75°	85°	20°	40°	90°	90°	45 kg/118%
11	65°	50°	15°	30°	90°	90°	40 kg/74%
12	75°	75°	20°	20°	90°	60°	28 kg/100%

^a^Absolute value and in comparison to the uninjured contralateral side

One patient developed a complex regional pain syndrome that was treated with oral medication and occupational therapy. At the final follow-up this patient presented a grip strength of 67% and a range of motion of 61% compared to the contralateral side.

The DASH and PRWE scores showed a mean of 24.2 (SD 25.1) and 32.2 (SD 29.4) points, respectively.

All but one patient specified a satisfaction rate of more than 5 with a mean value of 7.8 (SD 2.5).

MRI showed a dislocation (*n* = 7) or subluxation (*n* = 5) of the ECU tendon preoperatively. Five patients showed an unchanged displacement pattern postoperatively. However, four of them had an excellent and one a good Krimmer score and the satisfaction rate was between 8 and 10. One postoperative MRI was not assessable due to metallic artefacts after shorting osteotomy and osteosynthesis of the ulna styloid process.

## Discussion

The main finding of the study was that patients treated with a radially based extensor retinaculum flap for ECU tendon displacement showed a good to excellent function with a high satisfaction, grip strength and range of motion in comparison to the uninjured side. Good and excellent results could also be achieved even if the ECU tendon showed further instability in the postoperative MRI investigations.

This arises the question whether the instability is the main reason for complains or some other underlying problem, e.g. a potentially concomitant or subsequent irritating tendinosis or tenosynovitis, which is simultaneously treated intentionally or unintentionally. Jeantroux et al. showed that ECU tendinosis or tenosynovitis was present in all 16 investigated patients with a sub-sheath lesion [18]. Experience has shown that an unstable ECU tendon remained unstable for as long as the sub-sheath has not completely healed and this instability is thought to be the source of tenosynovitis [19].

Yet, several investigators have shown various ECU tendon displacement rates in asymptomatic volunteers [4, 5, 20–22]. Complete dislocation was seen in up to 39.5% [22]. Therefore, potentially displacement itself does not seem to be the source of complains but a potentially new additional lesion to the sub-sheath might trigger patient’s complains. It remains essential to correlate the radiologic findings with the clinical examination to correctly diagnose symptomatic ECU tendon instability. The “extensor carpi ulnaris synergy test”, “extensor carpi ulnaris displacement test” and the “heart-like test” might differentiate between an ECU tendinitis and ECU dislocation as the source of complaints [23].

Moreover, because the ECU tendon sheath is connected to the TFCC [24, 25], any lesion to this TFCC-sub-sheath binding or the TFCC itself might cause complains, which might mimic complains of an instable ECU tendon. Since any ECU tendon displacement might have been present before injury as seen in asymptomatic patients [4, 5, 20–22], an instable ECU tendon might be, potentially wrongly, regarded as the causative pathology for complains. TFCC and distal radioulnar joint pathologies can cause ulnar-sided wrist pain, which can be differentiated from an unstable ECU tendon by clinical examination and radiologic visualization using CT and MRI [23, 26–28].

Conservative treatment is proposed for acute lesions causing ECU tendon instability [7–10]. Five patients (tennis players) could be treated conservatively with a below-elbow cast fixation and 15° wrist extension for 2–4 months with a regular static MRI and ultrasound investigation and a stress test after 2 months. Return to competition was possible 5–6 months after injury without any symptoms [19].

However, some authors suggest conservative treatment only for acute type C lesions (avulsions of the periosteum) [11] or consider surgical treatment for acute cases due to the inadequate potential for anatomic healing of the fibrous sheath [12]. It is unknown which lesions might heal with conservative treatment [11].

Clinical distinguishing between the types of lesions is impossible [11], but MRI might show the exact site of the sub-sheath lesion [18]. While some authors classified all their surgically treated patients as a type C (stripping of the periosteum) [3], other investigators showed a distribution of 42–56% for type A lesions, 6.3–25% for type B lesions and 33–37% for type C lesions [11, 18].

Regarding surgical treatment, various procedures have been reported, however, there is no study comparing one technique to another.

The first describer of a similar procedure as in the present study showed no recurrent ECU dislocation in 3 patients with a follow-up of 2 years. This technique was said to allow normal rotatory range of motion [13].

A cohort of 12 patients (follow-up 6–48 months) showed a normal range of motion as well as a good and pain-free status at their previous work and sports activities after either a direct repair, a reconstruction with a free or pediculated flap. The treatment procedure was depended on the lesion type. There were no cases of recurrent dislocations. The authors stated that careful inspection of the lesion at the time of surgery should determine the type of repair [11].

A free extensor retinaculum flap showed good to excellent results in 9 patients with a mean follow-up of 23 months. Wrist mobility showed a deficit of 0–15° and grip strength was 70–100%. All patients were pain-free and returned to their previous activities without any clinical redislocation episode [15].

A fascia lata flap showed a subjective very good range of motion in 18 patients, a good in 4 patients and a moderate in 1 patient. Grip strength was subjectively slightly reduced in 5 patients and not reduced in 19 patients. Seventeen patients did not have subjective functional limitations, 3 had slight and 3 moderate limitations. There was no recurrence of clinical ECU tendon dislocation. Clinical testing showed a very good ECU tendon gliding in 7 patients, a good in 13, a moderate in 1 and a poor in 1 patient [16].

MacLennan et al. [3] reported on 21 patients with a type C injury [11] treated with a subperiosteal tendon sheath reconstruction and a groove deepening (follow-up 24–45 months). Range of motion, grip strength, satisfaction and the DASH score were significantly increased and pain decreased postoperatively. However, absolute changes for the range of motion and grip strength were low and did not seem clinically relevant [29]. Moreover, biomechanical testing showed that groove deepening did not improve ECU tendon stability in a cadaveric model compared with a sub-sheath reconstruction. A reconstruction was sufficient enough to eliminate dislocation events [30].

Another surgical treatment option, which was presented as a case report, is a potential release of the extensor retinaculum. The advantage is a faster rehabilitation without the need for long-term casting. The disadvantage is a bowstringing of the tendon with potential subsequent reduction of wrist extension and extension strength. However, the presented patient was painless, did not show any decreased range of motion or grip strength, had no snapping and could restart playing golf without any problems [17].

Less gratifying results are presented in six patients treated surgically without detailed procedure specifications. One patient showed a symptom-free recurrence after 18 months but improved his tennis ranking. Another person showed strongly disturbing scar formation and was unable to regain the previous tennis ranking. Two other patients showed fatigue pain. Worse results were potentially achieved because of a higher wrist demand of the presented competitive sports persons [20].

Due to inconsistently presented outcome parameters, comparisons for different techniques in the literature are difficult. No technique seems to be superior to another. The present study could show very good clinical results for the pediculated extensor retinaculum flap despite persistent tendon displacement in symptomatic patients with preoperatively ECU tendon subluxation and dislocation diagnosed using a rotation-movie MRI of the injured wrist. Although most authors report that their patients had no recurrent dislocation, no objective measurements were presented in contrast to the present study.

There are several limitations to this study besides its retrospective character. Preoperative data of all evaluated parameters are missing. Therefore, conclusions on improvements cannot be adequately reported. Moreover, because of this rare condition, the number of patients is low. At last, there was no other surgical procedure performed to which results could be compared to.

In conclusion, the pediculated extensor retinaculum flap as a treatment for a symptomatic ECU instability shows good to excellent results and a high subjective satisfaction independent of postoperative ECU tendon displacement.
